# Evaluating the process of care for persons admitted to Toronto area hospitals with acute severe ulcerative colitis

**DOI:** 10.1093/jcag/gwaf009

**Published:** 2025-05-28

**Authors:** Nasruddin Sabrie, Sonya Vukovic, Xin You, Surain Roberts, Fahad Razak, Amol A Verma, Laura E Targownik

**Affiliations:** Department of Medicine, University of Toronto, Toronto, M5S 1A8, Ontario, Canada; Department of Medicine, University of Toronto, Toronto, M5S 1A8, Ontario, Canada; St. Michael’s Hospital, Unity Health, Toronto, M5B 1W8, Ontario, Canada; St. Michael’s Hospital, Unity Health, Toronto, M5B 1W8, Ontario, Canada; Institute of Health Policy, Management and Evaluation, University of Toronto, Toronto, M5T 3M6, Ontario, Canada; Department of Medicine, University of Toronto, Toronto, M5S 1A8, Ontario, Canada; St. Michael’s Hospital, Unity Health, Toronto, M5B 1W8, Ontario, Canada; Institute of Health Policy, Management and Evaluation, University of Toronto, Toronto, M5T 3M6, Ontario, Canada; Department of Laboratory Medicine and Pathobiology, University of Toronto, Toronto, M5S 3K3, Ontario, Canada; Department of Medicine, University of Toronto, Toronto, M5S 1A8, Ontario, Canada; St. Michael’s Hospital, Unity Health, Toronto, M5B 1W8, Ontario, Canada; Institute of Health Policy, Management and Evaluation, University of Toronto, Toronto, M5T 3M6, Ontario, Canada; Department of Laboratory Medicine and Pathobiology, University of Toronto, Toronto, M5S 3K3, Ontario, Canada; Department of Medicine, University of Toronto, Toronto, M5S 1A8, Ontario, Canada; Division of Gastroenterology, Mount Sinai Hospital, Toronto, Ontario, Canada

**Keywords:** ulcerative colitis, quality improvement, process of care

## Abstract

**Background:**

Acute severe ulcerative colitis (ASUC) is associated with significant morbidity. In patients with ulcerative colitis (UC), the estimated lifetime risk of developing severe colitis is 25%. Several gastrointestinal societies have provided recommendations on pathways of care for managing ASUC. The degree to which they are adhered to in different care settings remains unclear.

**Methods:**

We conducted a retrospective review using data from 7 acute-care hospitals collected through the general medicine inpatient initiative (GEMINI), a hospital research collaborative that collects administrative and clinical data from hospital information systems. We identified all patients with the most responsible inpatient discharge diagnosis of ulcerative colitis between April 2015 and December 2019. The primary outcome was the difference in hospital length of stay of patients admitted with ASUC based on hospital-type; community, academic, or inflammatory bowel disease (IBD)-focussed sites.

**Results:**

765 eligible patients were identified between April 2015 and December 2019. The mean hospital length of stay was 9.21 days for the academic sites, 6.94 days for the community sites, and 8.03 for the IBD specialty centre (*P* = .094). Adverse events were uncommon overall. In our multiple logistic regression analysis, we identified that admission to an IBD-focussed centre compared to an academic centre, carried an odds ratio of 2.07 (95% CI, 1.16-3.78) for the outcome of inpatient-colectomy.

**Conclusions:**

The processes of care for patients with ASUC varied on the basis of the type of hospital they were admitted to, with the IBD specialty centre providing the most guideline adherent care. Low-cost interventions should be utilized to promote adherence to clinical practice recommendations.

## Background

Acute severe ulcerative colitis (ASUC) is associated with significant morbidity^1^ and is defined as worsening of inflammatory activity requiring hospitalization for monitoring or therapy. In patients with ulcerative colitis (UC), the estimated lifetime risk of developing severe colitis is 25% with approximately 10%-15% of patients presenting with ASUC at diagnosis.^1,2^ The initial approach to ASUC consists of medical management with hydration, pain control, and medications to reduce the inflammatory burden. However, failure of medical management is a frequently encountered scenario with colectomy being required in as many as 27%-30% of patients.^3^ Persons with ASUC are also at increased risk of in-hospital complications, including venous thromboembolism, hospital-acquired infections, superimposition of *Clostridioides difficile* infection (CDI), and malnutrition.^4^

In an effort to encourage the provision of standardized high-quality evidence-based care of ASUC, the American College of Gastroenterology (ACG), the American Gastroenterological Association (AGA), and the Canadian Association of Gastroenterology (CAG) have provided guidelines on recommended pathways of care.^4–6^ These guidelines recommend screening for CDI on presentation and ensuring the provision of thromboembolic prophylaxis. Recommendations also include treating patients with intravenous steroids, with a transition to either infliximab, other biologics or cyclosporine as rescue therapy for nonresponders. Guidelines further recommend monitoring the response to medical therapy both through assessing the clinical and symptomatic response and through serial measurement of C-reactive protein (CRP), with surgical consultation recommended for patients without a brisk response to medical interventions^4–6^ (Table 1).

**Table 1.** Guideline recommendations, and quality indicators, in managing patients with ASUC.

Quality indicators	Recommendation(s) from ACG/AGA/CAG	Method for evaluating adherence to recommendations
***Medications***		
Corticosteroids	• Intravenous corticosteroids (IVCS) recommended as first-line therapy (methylprednisolone 40-60 mg/day or equivalent) • Continued use of systemic steroids beyond 7 days not effective in nonresponders^1,2^	• Proportion of patients receiving IVCS within 72 h of admission • Proportion of patients receiving IVCS for 5 continuous days
VTE prophylaxis	• Recommend DVT prophylaxis	• Proportion of patients receiving low-molecular-weight heparin, low-dose heparin, or any other anticoagulant within 24 h of admission
Opioids	• Avoid use of narcotics	• Proportion of patients receiving opioids during their admission
Antibiotics	• Avoid routine antibiotic use without clear infection	• Proportion of patients receiving antibiotics during their admission
Immunosuppressant	• Cyclosporine recommended as second-line rescue therapy in steroid nonresponders	• Proportion of patients receiving noncorticosteroid immunosuppressants
Biologics	• Infliximab recommended as second-line rescue therapy in hospitalized steroid nonresponders • No specific dosage recommendation	• Proportion of patients receiving a biologic agent (including infliximab, adalimumab, certolizumab, golimumab, vedolizumab, and tofacitinib)
***Microbiology testing***		
* C. difficile* (stool)	*• *Recommend testing for *C.difficile* infection	• Not captured in reported data
Screening for latent infections (tuberculosis, hepatitis B)	• Recommend screening for latent infections	• Not captured in reported data
***Inflammatory Markers***		
CRP	• Day 3 CRP may be used as an objective measure in aiding to determine steroid responsiveness	• Proportion of patients with CRP measured within 48 h of admission • Proportion of patients that have been admitted for at least 3 days with repeated CRP at days 3-5
***Procedures***		
Endoscopy	• Recommended as early as possible in admission evaluating severity and testing for CMV and *C. difficile* colitis	• Proportion of patients receiving endoscopy during admission
Colectomy	• Recommended in rescue therapy nonresponders	• Proportion of patients receiving in-patient colectomy
***Complications***		
Intestinal perforation	• Not applicable	• Proportion of patients experiencing complications of ASUC
Toxic megacolon		
VTE		
* C. difficile* infection		
Death		

Despite the provision of these guidelines by gastroenterology societies, the degree to which they are adhered to in different care settings remains unclear. Studies have highlighted the presence of heterogeneity in the treatment of patients admitted with severe UC, even among expert providers.^7^ This variability is likely augmented at care centres with lower volumes of inflammatory bowel disease (IBD) patients and less IBD expertise.^8^ Although some degree of heterogeneity in management is expected, and reflects the unique circumstances patients face, findings of excess variation in the processes of care between different healthcare settings may herald disparities in the quality of care, which likely contribute to avoidable adverse events.^9^ The purpose of our study is to evaluate the processes of care and their associated outcomes in patients admitted with ASUC to the following different hospital settings in the Greater Toronto Area (GTA); IBD specialty sites, tertiary academic hospitals, and community hospitals.

## Methods

### Data source

We used data from 7 acute-care hospitals (5 academic and 2 community hospitals) in the GTA collected through the general medicine inpatient initiative (GEMINI). We considered a hospital to be either an academic site or a community site, in accordance with the classification of hospitals provided by the provincial Ministry of Health and Long-Term Care.^10^ GEMINI is a hospital research collaborative that collects administrative and clinical data from hospital information systems with 98%-100% accuracy of selected data elements when compared to manual chart review.^11^ GEMINI includes all adult admission to the department of medicine or intensive care unit from 31 hospitals from 20 healthcare organizations across Ontario.^11^ The academic sites included in our study were St Michael’s Hospital (SMH), Mount Sinai Hospital (MSH), Sunnybrook Health Sciences Centre (SBK), the University Health Network including Toronto General Hospital (TGH), and Toronto Western Hospital (TWH). In addition to being an academic site, MSH is also a regional IBD-specialty centre with a dedicated IBD ward and expertise in the collaborative medical and surgical care of patients with IBD. Community sites included in GEMINI for this study are Trillium Health Partners Credit Valley Hospital (THPC) and Mississauga Hospital (THPM). GEMINI contains administrative data that are reported by hospitals to the Canadian Institute of Health Information (CIHI) Discharge Abstract Database and National Ambulatory Care Reporting System,^12^ including but not limited to patient transfers and patient discharges. GEMINI also contains clinical data from electronic health records including but not limited to laboratory results, imaging reports and medication orders during hospitalizations. At the individual patient level, administrative health data, and clinical data extracted from hospital information systems were linked.

### Inclusion criteria

We identified all patients with the most responsible inpatient discharge diagnosis of ulcerative colitis (ICD-10-CA K51.X) who were admitted through the emergency department and admitted to or discharged from a general internal medicine (GIM) or gastrointestinal (GI) ward, between April 2015 and December 2019. Patients were excluded if they were under the age of 18 or listed as being pregnant based on ICD-10 code Z34.90, at the time of admission. Patients admitted directly from the emergency room to surgical wards were not included in our study, as direct surgical admissions are not captured by GEMINI.

### Data collection

Demographic data, including age and sex, were identified using the CIHI discharge abstract database, as were the date of admission, date of discharge, inter-hospital transfers, and inhospital deaths. Comorbidities including the Charlson comorbidity index scores were collected, as was the modified laboratory-based acute physiology score (mLAPS), a validated predictor of inpatient mortality.^13^ Neighborhood income quintile was identified using census data linked to the hospitalizations at the level of dissemination area. We used results from laboratory testing throughout the hospital stay, with attention to c-reactive protein (CRP), erythrocyte sedimentation rate (ESR), haemoglobin (Hgb), white blood cell count (WBC), platelets (Plt), albumin (Alb), and creatinine (Cr).

Medications were identified via manual mapping by a clinical subject matter expert (SV) and validated by 2 authors (L.T. and A.V.). All medications provided at the time of hospital admission and throughout hospitalization were considered, with particular attention paid to the use of intravenous and oral corticosteroids, IBD-related biologics (including infliximab, adalimumab, vedolizumab, and ustekinumab) and other IBD-related advanced therapies (tofacitinib and cyclosporine). We also assessed the in-hospital use of subcutaneous low-dose heparin and low molecular weight heparin for venous thromboembolism (VTE) thromboprophylaxis, and the use of opioids for pain control. Procedures during hospitalization including gastrointestinal endoscopy or colectomy were identified using Canadian Classification of Health Intervention (CCI) codes (Table S1). Adverse events were recorded including rates of *Clostridium difficile* infection, thromboembolic events, nontraumatic bowel perforation, inpatient colectomy, and death.

### Outcomes

The primary outcome was the difference in hospital length of stay of patients admitted with ASUC on the basis of hospital type. Key secondary outcomes included the following processes of care: proportion of patients receiving thromboembolism prophylaxis within 24 h of admission, proportion of patients initiated on corticosteroids and duration of steroids, proportion of patients with baseline CRP measurement and repeat CRP between day 3 and 5 of admission, proportion of patients remaining on intravenously (route) (IV) steroids on day 5 of admission, proportion of patients receiving biologics among those who remained on IV corticosteroids by day 5 and proportion of patients receiving opioids in hospital. We also determined the frequency of adverse events associated with UC hospitalizations, including the proportion of patients experiencing thromboembolism, *C. difficile* infection, inpatient colectomy, nontraumatic bowel perforation, and death.

### Statistical analysis

All statistical analysis was conducted in R version 4.2.0.^14^ Categorical variables were evaluated using the Chi-Square test and continuous variables were evaluated using either ANOVA or Kruskal-Wallis test based on their distributions. We used multivariable logistic regression to evaluate the adjusted association between hospital type and the following outcomes: (a) inhospital colectomy; (b) composite adverse events defined as experiencing 1 or more of *C. difficile* infection, thromboembolic event, nontraumatic bowel perforation, inpatient colectomy, or death. Logistic regressions were adjusted for age, sex, receipt of steroid on admission, and albumin level greater than 30g/L. Statistical significance was evaluated at an alpha of 0.05 and post hoc corrections were not employed for multiple comparisons.

## Results

### Baseline characteristics

765 eligible patients were identified between April 2015 and December 2019. 308 patients were admitted to general academic sites, 137 patients were admitted to community sites, and 320 patients were admitted to an IBD-focussed academic centre (Figure S1).

Patient characteristics, by hospital type, are highlighted in Table 2. Patients admitted to the IBD centre were younger, were more often female, and were more commonly from the highest income quintile neighbourhoods, compared to patients admitted to general academic or community sites. Patients from all 3 hospital types had similar mLAPS scores and over 90% of all patients had a Charlson score of 0 at admission. The most common comorbidities captured were hypertension (4%) and diabetes (7%). Laboratory data were similar between all 3 cohorts, however, patients admitted to an IBD-focussed centre were less likely to have albumin of less than or equal to 30 g/L (18.8%), compared to patients admitted to academic (30.8%) or community sites (29.9%), respectively.

**Table 2.** Baseline characteristics of included patients by hospital type.

	Academic site *N* = 308	Community site *N* = 137	IBD specialty centre *N* = 320	Overall *N* = 765
**Sex**				
Female	137 (44%)	64 (47%)	168 (52%)	369 (48%)
Male	171 (56%)	73 (53%)	152 (48%)	396 (52%)
**Age (mean, SD)**	43 (±19)	47 (±20)	35 (±14)	40 (±18)
**Disposition**				
Transfers from other hospital	0 (0%)	0 (0%)	<6 obs.	<6 obs.
Nonhome discharge^b^	19 (6%)	<6 obs.	13 (4%)	36 (5%)
**Neighborhood income** ^a^				
1	67 (22%)	32 (23%)	54 (17%)	153 (20%)
2	65 (21%)	30 (22%)	63 (20%)	158 (21%)
3	55 (18%)	32 (23%)	51 (16%)	138 (18%)
4	43 (14%)	21 (15%)	65 (20%)	129 (17%)
5	67 (22%)	20 (15%)	82 (26%)	169 (22%)
Missing	11 (3.6%)	2 (1.5%)	5 (1.6%)	18 (2.4%)
**Charlson score at admission**				
0	285 (93%)	126 (92%)	314 (98%)	725 (95%)
1	12 (4%)	<6 obs.	<6 obs.	19 (2%)
2	10 (3%)	7 (5%)	<6 obs.	19 (2%)
3	0 (0%)	<6 obs.	0 (0%)	<6 obs.
5	<6 obs.	0 (0%)	0 (0%)	<6 obs.
**Modified LAPS score**				
Mean (SD)	13 (±12)	12 (±12)	11 (±11)	12 (±11)
**Comorbidities**				
Hypertension	12 (4%)	< 6 obs.	11 (3%)	27 (4%)
Diabetes	18 (6%)	16 (12%)	19 (6%)	53 (7%)
Coagulopathy	<6 obs.	0 (0%)	<6 obs.	<6 obs.
Malignancy	<6 obs.	<6 obs.	<6 obs.	7 (1%)
**Laboratory data**				
CRP ≥ 11	206 (66.9%)	85 (62.0%)	217 (67.8%)	508 (66.4%)
ESRP (mean, SD)	38.8 (24.9)	39.8 (27.8)	36.8 (23.3)	37.9 (24.5)
Leukocytes (mean, SD)	11.5 (5.57)	11.5 (6.85)	11.3 (5.17)	11.4 (5.59)
Haemoglobin (mean, SD)	120 (26.4)	121 (21.8)	119 (22.5)	119 (24.0)
Creatinine (mean, SD)	81.4 (42.8)	94.8 (70.5)	71.7 (21.5)	79.8 (43.5)
Albumin (g/L) ≤ 30	95 (30.8%)	41 (29.9%)	60 (18.8%)	196 (25.6%)
Platelets (mean, SD)	366 (146)	362 (146)	376 (133)	369 (141)
**Receipt of corticosteroid** ^c^	197 (64%)	86 (63%)	249 (78%)	532 (70%)
**Receipt of IV corticosteroid** ^c^	168 (55%)	78 (57%)	213 (67%)	459 (60%)

Variables with fewer than 6 observations were suppressed per mandated privacy guidelines. Post-hoc corrections were not employed for multiple comparisons.

^a^Neighborhood income quintiles range from 1 (lowest income quintile) to 5 (highest income quintile).

^b^Refers to discharge to a skilled nursing facility, rehabilitation facility and/or separate acute care unit.

^c^Receipt of steroid within 72 h of admission.

### Process of care measures of patients admitted with ASUC

The results of our key process of care measures are summarized in Table 3. The mean hospital length of stay was 9.21 days for the academic sites, 6.94 days for the community sites, and 8.03 for the IBD specialty centre (*P*-value = .094). The rate of baseline CRP measurement was different between the 3 cohorts with 81% of patients from academic sites, 76% of patients from community sites, and 95% of patients from an IBD-focussed site having baseline values recorded (*P*-value < .001) (Table 3). Of patients with at least a 5-day hospital length of stay, 39% of patients in academic sites, 75% of patients from community sites, and 47% of patients from the IBD-focussed centre were on intravenous steroids for 5 consecutive days (*P*-value < .001). Patients admitted to academic hospitals (22%) and community hospitals (23%) were less likely to receive a biologic during their hospital stay compared to patients admitted to the IBD-focussed site (37%) (*P*-value < .001). Adverse events were overall uncommon with *C. difficile* infection occurring in 1% of all patients and colectomy occurring in 8% of all patients (Table 4).

**Table 3.** Hospital length of stay (HLOS) and processes of care for patients admitted with ASUC.

Process of care	Academic site *N* = 308	Community site *N* = 137	IBD specialty centre *N* = 320	Overall *N* = 765	*P*-value
**Mean (SD) HLOS (days)**	9.21 (13.4)	6.94 (7.39)	8.03 (8.34)	8.31 (10.6)	.094
**Baseline CRP measured** ^a^	251 (81%)	104 (76%)	305 (95%)	660 (86%)	**<.001**
**Day 3-5 CRP measured** ^c^	113 (47%)	51 (52%)	129 (50%)	293 (49%)	.590
**Receipt of low molecular weight heparin** ^b^	128 (42%)	51 (37%)	252 (79%)	431 (56%)	**<.001**
**Receipt of intravenous corticosteroids for **≥**5 days**^d^	73 (39%)	49 (75%)	94 (47%)	216 (48%)	**<.001**
**Receipt of biologic**	68 (22%)	31 (23%)	117 (37%)	216 (28%)	**<.001**
**Receipt of biologic while on day 5 of corticosteroids**	47 (48%)	26 (57%)	76 (58%)	149 (54%)	.27
**Use of opioids**	134 (44%)	64 (47%)	149 (47%)	347 (45%)	.699

^a^CRP measured within 48 h of admission.

^b^Receipt of venous thromboembolism prophylaxis within 24 h of admission.

^c^Proportion of patients with at least 3 days length of stay.

^d^Proportion of patients with at least 5 days length of stay.

**Table 4.** In-hospital adverse events of patients admitted with ASUC.

Adverse events	Academic site *N* = 308	Community site *N* = 137	IBD specialty centre *N* = 320	Overall *N* = 765	*P*-value
**Venous thromboembolism**	<6 obs.	<6 obs.	<6 obs.	<6 obs.	.830
***C. difficile* infection**	<6obs.	0 (0%)	<6 obs.	9 (1%)	.418
**Colectomy**	20-25 (7%)	<6 obs.	37 (12%)	63 (8%)	**.006**
**Bowel perforation**	<6 obs.	0 (0%)	<6 obs.	<6 obs.	1.000
**Death**	<6 obs.	0 (0%)	0 (0%)	<6 obs.	.488

### Multiple logistic regression for colectomy and adverse events

In the multivariable logistic regression analysis, admission to an IBD-focussed centre was associated with the outcome of inpatient-colectomy, compared to academic centres (OR 2.07, 95% CI, 1.16-3.78) (Table 5). Conversely, for the composite adverse event outcome, admission to an IBD-focussed centre was not a statistically significant variable in our regression model (odds ratio = 0.95, 95% CI, 0.33-2.70) (Table 6).

**Table 5.** Multiple logistic regression modelling colectomy in patients admitted with ASUC.

Predictor variables	Odds ratio	95% CI	*P*-value
Age	1.02	1.00-1.03	**.04**
Male	1.09	0.64-1.85	.76
Receipt of steroids on admission	1.81	1.04-3.27	**.04**
Albumin > 30 g/L^a^	0.48	0.28-0.85	**.01**
Admission at community hospital^b^	0.40	0.11-1.08	.10
Admission at IBD centre^b^	2.07	1.16-3.78	**.02**

*P* < 0.05.

^a^Missing albumin values were imputed as normal (40 g/L).

^b^Reference variable is academic sites.

**Table 6.** Multiple logistic regression modelling composite adverse events in patients admitted with ASUC.

Predictor variables	Odds ratio	95% CI	*P*-value
Age	1.02	0.99-1.04	.23
Male	0.70	0.26-1.83	.46
Receipt of steroids on admission	0.67	0.24-1.80	.43
Albumin > 30 g/L^a^	0.23	0.08-0.61	**.004**
Admission at community hospital^b^	0.19	0.01-1.04	.12
Admission at IBD centre^b^	0.95	0.33-2.70	.93

*P* < 0.05.

^a^Missing albumin values were imputed as normal (40 g/L).

^b^Reference variable is academic sites. Composite adverse events included venous thromboembolism, *C. difficile* infection, colectomy, bowel perforation, or death.

## Discussion

In our retrospective multicentre study of patients admitted to general medicine or gastroenterology wards with acute, severe ulcerative colitis, and adherence to clinical practice recommendations was variable between the IBD specialty centre, academic hospitals, and community hospitals. Patients admitted to the IBD specialty centre were more likely to receive baseline CRP measurement, venous thromboembolism prophylaxis, and treatment with intravenous steroids and biologic therapy during their admission. Patients admitted to the IBD specialty site were more likely to receive a colectomy during their admission.

Adherence to clinical practice guidelines within IBD has been reported to be inconsistent. In a cross-sectional study assessing compliance with the AGA’s quality metrics for care of patients with IBD in the outpatient setting, overall compliance was noted to be below expectation with only 6.5% of patients having all core measures of quality evaluated and documented.^15^ Beyond IBD, processes of care have also been reported to be inconsistent between academic and community settings, across various clinical contexts. The extent to which this variability directly results in adverse outcomes is unclear. In a retrospective cohort study of 1000 endoscopic retrograde cholangiopancreatographies (ERCP) performed for choledocholithiasis, nonadherence to guideline-directed indications for ERCP in suspected choledocholithiasis was significantly higher in community settings compared to academic settings.^16^ This nonadherence likely contributed to the higher rate of delay in care and harm in patients treated at community sites.^16^ Patel et al., demonstrated a similar discordance in adherence to clinical practice guidelines for management of non-ST elevation myocardial infarction, between academic and community sites, with academic hospitals demonstrating a higher composite adherence to recommendations.^8^ Although adverse inhospital events were similar between the two clinical settings, the authors highlight a higher degree of variation in adherence to clinical practice guidelines, in nonacademic community hospitals.^8^

In the Donabedian model for evaluating the quality of care, patient outcomes are determined by both, the structure that care is delivered in, and the specific processes involved in providing such care.^17^ In our study, we identified that specific processes associated with high-quality care for IBD patients with ASUC occurred at a higher rate at the IBD-focussed centre, compared to academic or community sites. The substantial structural differences between these hospital types, including differences in clinicians and surgical expertise in managing IBD and the availability of dedicated inpatient IBD units, may result in knowledge gaps about the existence and content of clinical practice guidelines in managing ASUC. These factors likely contribute to the disparity we observed between hospital types in achieving key quality metrics. However, compared to structural differences, processes of care are more malleable and are responsive to quality improvement initiatives, often at low cost. For example, in an effort to increase the proportion of immunosuppressed IBD patients who receive appropriate vaccinations, a quality improvement process was implemented which involved restructuring the patient’s clinical encounters to begin with the completion of a vaccine questionnaire. This was followed by receipt of the appropriate vaccination after being seen by their clinician.^18^ This simple intervention resulted in substantial improvement in rates of influenza and pneumococcal vaccination in this population compared to the previous 5 years.^18^

Despite our findings that favour the IBD-specialty site as one with higher quality and guide-line adherent care, our multiple logistic regression analysis revealed the odds of receiving a colectomy as an inpatient to be twice as much for patients admitted to an IBD centre, compared to patients admitted to a community or academic site. There are 2 possible explanations for this finding. Firstly, patients cared for at the IBD centre often have a more complex IBD history with multiple lines of therapy attempted and are often referred from peripheral clinics for more specialized care. Consequently, these patients are likely at higher risk for medical failure requiring colectomy. The clinical characteristics we were able to report do not provide us with sufficient information to characterize this difference as we were not able to measure specific pre-admission IBD medications, prior biologic failure or the duration of ulcerative colitis, thus likely underestimating this contrast in complexity between the 2 groups. Secondly, the IBD specialty centre comprises not only gastroenterologists with a focus on IBD but colorectal surgeons that work closely in tandem and offer surgery for medically refractory disease. Thus, it is likely that the collaborative relationship between gastroenterology and colorectal surgery, coupled with the expertise offered at this site, allows for colectomy to be considered sooner. There is evidence that early surgical involvement in patients with ASUC is beneficial. In a study of 2650 patients who underwent nonelective colectomies, patients fared better, with a 46% reduction in rates of complication, when surgery was performed earlier.^19^

Our study provides insight into an important knowledge gap in managing ulcerative colitis and highlights the heterogeneity of care provided over a large catchment area. Despite the strengths of our study, we report several limitations. Firstly, our data did not capture all components of the Truelove–Witts criteria for ASUC,^20^ including bowel movement frequency and degree of rectal bleeding, thus it is possible that our study included patients not fulfilling this criterion and to whom the recommended processes of care may not apply. Secondly, the pre-admission severity and complexity of a patient’s IBD history, specifically their pre-admission IBD medications, biologic use history, and duration of disease, was not measured which limits our ability to delineate patients with severe baseline disease from those with more stable disease. Thirdly, GEMINI does not capture patients admitted to surgical services. Consequently, our study may underestimate colectomy rates and not capture patients with fulminant colitis on presentation requiring immediate surgery. By not capturing these admissions we are likely also underestimating adverse events, including thromboembolism, infection, and death. Additionally, our data collection precedes significant changes in the landscape of medical management of ASUC, such as the use of Janus kinase inhibitors (JAKi). As such, some of the differences or lack thereof, between IBD centres and non-IBD centres may not necessarily be to the same degree. For example, it is plausible that in anti-TNF-exposed patients with ASUC, the duration of intravenous corticosteroids may be reduced in centres with access to JAKi. Furthermore, reliance on administrative data, which typically involves using data that was not collected for the specific research question, may carry the risk of misclassification bias, unmeasured confounding, and missing data. Finally, this study relied on retrospective data, which is prone to bias.^21^

## Conclusions

The processes of care for patients with ASUC varied on the basis of the type of hospital they were admitted to, with the IBD specialty centre providing the most guideline adherent care. Low-cost interventions should be utilized to bridge this gap between institutions and promote adherence to clinical practice recommendations.

## Supplementary material

Supplementary material is available at *Journal of the Canadian Association of Gastroenterology* online.

gwaf009_suppl_Supplementary_Tables_S1

gwaf009_suppl_Supplementary_Figures_S1

gwaf009_suppl_Supplementary_ICMJE

## Data Availability

The de-identified data may be shared on reasonable request to the corresponding author should the request comply with IRB requirements and institutional policy.
