# Supplementary material for: Evaluating the process of care for persons admitted to Toronto area hospitals with acute severe ulcerative colitis
Source: J Can Assoc Gastroenterol. 2025 May 28;8(4):120–7. doi: 10.1093/jcag/gwaf009 (PMC12401000; doi:10.1093/jcag/gwaf009)
Supplement: gwaf009_suppl_Supplementary_Tables_S1 [file gwaf009_suppl_supplementary_tables_s1.docx]

**SUPPLEMENTARY**

**Table S1**

**Canadian Classiﬁcation of Health Interventions (CCI)**

| NM.87, NM.89, NM.91, NQ.87 | Colectomy |
| --- | --- |
| NM.70, NQ.70 | Gastrointestinal endoscopy |

**Table S2**

**International Classification of Diseases, Tenth Revision, Canada (ICD-10-CA)**

| K51.x | Ulcerative Colitis |
| --- | --- |
| I10.x | Hypertension |
| I09.9, I11.0, I13.0, I13.2, I25.5, I42.0, I42.5 - I42.9, I43.x, I50.x, P29.0 | Congestive heart failure |
| G45.x, G46.x, I60.x - I63.x, I65.x-I68.x | Cerebrovascular disease |
| E10.x, E11.x, E13.x | Diabetes |
| C00.x - C26.x, C30.x - C34.x, C37.x - C41.x, C43.x, C45.x - C58.x, C60.x - C76.x, C81.x - C85.x, C88.x, C90.x - C97.x | Any malignancy, including lymphoma and leukemia, except malignant neoplasm of skin |
| D65 - D68.x, D69.1, D69.3 - D69.6, D69.8, D69.9 | Coagulopathy |
| F33.0- F33.4, F33.8, F33.9 | Depression |
