# Supplementary figures and images for: Evaluating the process of care for persons admitted to Toronto area hospitals with acute severe ulcerative colitis
Source: J Can Assoc Gastroenterol. 2025 May 28;8(4):120–7. doi: 10.1093/jcag/gwaf009 (PMC12401000; doi:10.1093/jcag/gwaf009)

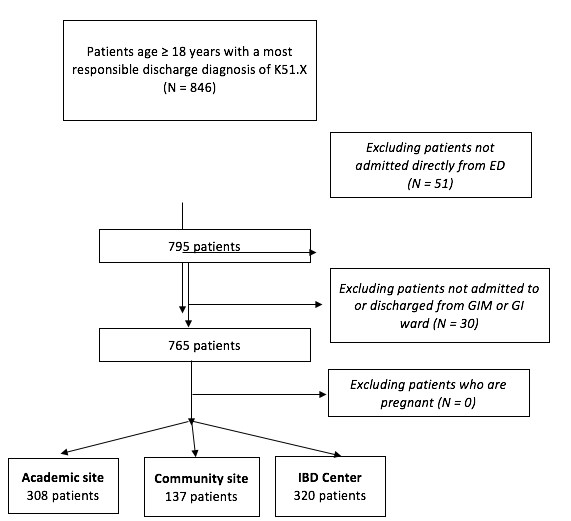

Supplement: gwaf009_suppl_Supplementary_Figures_S1 [file gwaf009_suppl_supplementary_figures_s1.jpeg]
